# Cross-Talk Between the Intestinal Epithelium and *Salmonella* Typhimurium

**DOI:** 10.3389/fmicb.2022.906238

**Published:** 2022-06-06

**Authors:** Sandrine Ménard, Sonia Lacroix-Lamandé, Katrin Ehrhardt, Jin Yan, Guntram A. Grassl, Agnès Wiedemann

**Affiliations:** ^1^IRSD - Institut de Recherche en Santé Digestive, Université́ de Toulouse, INSERM, INRAE, ENVT, UPS, Toulouse, France; ^2^ISP, INRAE, Université de Tours, Nouzilly, France; ^3^Institute of Medical Microbiology and Hospital Epidemiology, Hannover Medical School and German Center for Infection Research (DZIF), Hannover, Germany; ^4^Department of Gastroenterology, The Second Xiangya Hospital of Central South University, Changsha, China; ^5^Research Center of Digestive Disease, Central South University, Changsha, China

**Keywords:** gastrointestinal tract, bacteria, invasion, survival, host defense

## Abstract

*Salmonella enterica* serovars are invasive gram-negative bacteria, causing a wide range of diseases from gastroenteritis to typhoid fever, representing a public health threat around the world. *Salmonella* gains access to the intestinal lumen after oral ingestion of contaminated food or water. The crucial initial step to establish infection is the interaction with the intestinal epithelium. Human-adapted serovars such as *S.* Typhi or *S.* Paratyphi disseminate to systemic organs and induce life-threatening disease known as typhoid fever, whereas broad-host serovars such as *S.* Typhimurium usually are limited to the intestine and responsible for gastroenteritis in humans. To overcome intestinal epithelial barrier, *Salmonella* developed mechanisms to induce cellular invasion, intracellular replication and to face host defence mechanisms. Depending on the serovar and the respective host organism, disease symptoms differ and are linked to the ability of the bacteria to manipulate the epithelial barrier for its own profit and cross the intestinal epithelium.

This review will focus on *S.* Typhimurium (STm). To better understand STm pathogenesis, it is crucial to characterize the crosstalk between STm and the intestinal epithelium and decipher the mechanisms and epithelial cell types involved. Thus, the purpose of this review is to summarize our current knowledge on the molecular dialogue between STm and the various cell types constituting the intestinal epithelium with a focus on the mechanisms developed by STm to cross the intestinal epithelium and access to subepithelial or systemic sites and survive host defense mechanisms.

## Introduction

*Salmonella enterica* serovars are prevalent human and animal pathogens responsible for gastroenteritis and typhoid disease. The typhoid fever is mainly caused by *S. enterica* serovar Typhi (*S.* Typhi), while non-typhoidal *Salmonella* strains such as the broad-host serovars Typhimurium (*S.* Typhimurium; STm), normally cause salmonellosis, known as gastroenteritis. STm is one of the most common bacterial pathogens worldwide, causing 109.9 Million cases of food poisoning per year (Stanaway et al., 2016) and this serovar is the object of this study. In immunocompetent persons salmonellosis is usually self-limiting but immune-depressed persons, the elderly and young children may develop severe complications leading to sepsis and death (Feasey et al., 2012).

STm is mainly spread by contaminated animal-derived food products such as egg and chicken, or fresh produce and water contaminated with feces from infected hosts (Velge et al., 2012). Following oral ingestion, STm reaches the gastrointestinal tract in mammals, where the bacterium is able to attach and invade the intestinal epithelium allowing intracellular bacterial replication and colonization. In addition, STm can spread systemically, leading to bloodstream infection and dissemination to deep organs such as the spleen and the liver. The interaction with the intestinal epithelium is thus a crucial step for the establishment of STm infection. However, no clear data are available to identify if STm target a specific intestinal epithelial cell (IEC) type. Indeed, even though numerous studies performed in intestinal epithelial cell lines (enterocytes) provided data on molecular mechanisms regarding entrance of STm on IECs, they are limited to one epithelial cell type and their relevance *in vivo* is still under debate. Organoid models represent an interesting tool to fill those gap, but studies are still scarce and limitations have been described.

In this review, we aimed to summarize and discuss the knowledge regarding interactions between STm with each intestinal epithelial cell in available models *in vitro* and *in vivo*. The review is structured as follow: (i) description of epithelial cells and associated functions present in the intestine; (ii) the role of barrier of intestine; (iii), the listing of mouse model available for STm infection; (iv) the knowledge on STm entrance in each cell type of the intestinal epithelium; (v) description of the defence responses of the intestinal epithelium toward STm; (vi) the interest of organoid models to study STm interaction with IECs. This review draws an overview of the knowledge on STm interaction with every IECs based on *in vitro* and *in vivo* human and mouse models.

## Composition of the Intestinal Epithelium

**Figure 1:**
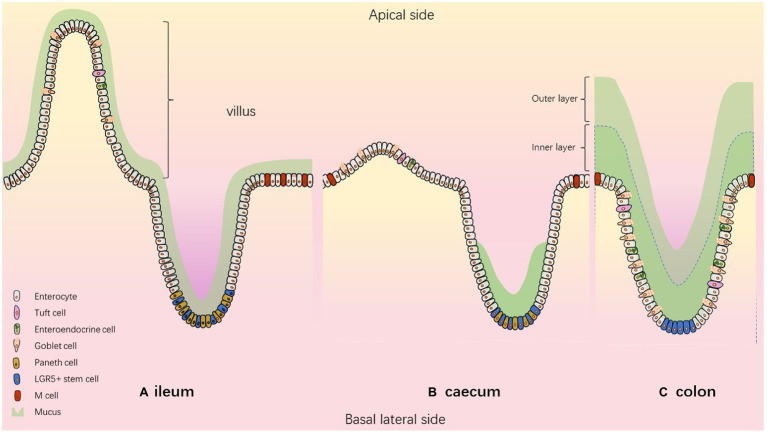
The composition of the intestinal epithelium according to the segment. Model describing **(A)** ileum **(B)** caecum **(C)** colon.

The intestinal tract fulfils the dual function of nutrient absorption and defence against pathogens. Indeed, the intestine has to absorb nutrients from food chime and prevent pathogen entrance (bacteria, virus, and parasites). To achieve those goals, the intestine is regionalized and composed of specialized cell types. The intestinal tract is composed of the small and large intestine mainly devoted to nutrient and water absorption, respectively. The small intestine is then divided, from proximal to distal part, in duodenum, jejunum and ileum and the large intestine is composed of caecum and colon (Figure 1). Human and mice present similarities in intestinal architecture despite the appendix in human representing a vestige of the caecum much more developed in mice. Thanks to various levels of folds and invaginations, the intestinal epithelium is the largest surface in contact with the outside.

The intestinal barrier is composed of epithelial stem cells, transit amplifying cells and highly specialized epithelial cells including absorptive cells (enterocytes, Microfold-M cells) and secretory cells (enteroendocrine, goblet, tuft and paneth cells; Elmentaite et al., 2021). All these cell types are generated from intestinal stem cells (ISC) located near the bottom of the crypts. ISCs continually proliferate, and newly formed cells migrate along the crypt-villus axis where they differentiate and acquire specialized functions. After 3–5 days of upward migration, differentiated cells reach the top of the villi, where they are shed into the gut lumen and die by anoïkis (Figure 1).

### Enterocytes

Enterocytes are polarized cells which are more numerous in the small intestine than in the caecum and colon and represent more than 80% of all epithelial cells. Their function is to absorb molecules from the lumen and transport them to the bloodstream. The enterocytes of the small intestine absorb most of the nutrients, whereas the absorption of water mainly occurs through the large intestinal enterocytes called colonocytes (Han et al., 2021; Aguirre Garcia et al., 2022).

### Goblet Cells

Goblet cells produce mucus-filled granules that are secreted into the lumen to protect the mucosa. The mucus is mainly composed of glycosylated proteins called mucins. It forms a gelatinous hydrogel, blocking the contact between the epithelium and microorganisms such as bacteria. The architecture of the mucus differs according to the intestinal segment, and the thickness of this mucus layer depends on several variables including the composition of the microbiota, the host species and diet (Jakobsson et al., 2015). The mucus in the distal colon is constantly renewed by goblet cells (Johansson, 2012) and is composed of a mostly sterile inner layer and an outer layer, colonized by bacteria (Johansson et al., 2008; Li et al., 2015). In the ileum, a continuous very thin and easily detachable mucus layer has been described. In the caecum, a continuous mucus layer is missing. Only the bottom of the intestinal crypts is filled by dense mucus, leaving the cells between crypts accessible to microorganisms (Atuma et al., 2001; Ermund et al., 2013; Furter et al., 2019). However, a new organization of colonic mucus has been proposed recently, demonstrating that mucus layer in distal colon covers colonic content but not colonic epithelium when the lumen is empty (Kamphuis et al., 2017).

### Enteroendocrine Cells

Enteroendocrine cells have a tall and columnar appearance. Microvilli are present at their apical surface and the cytoplasm contain granules, containing peptide hormones that are secreted from the basal side of the epithelium and act in a paracrine or endocrine manner (Yen and Wright, 2006). They are found scattered throughout the entire length of the intestine in both crypts and villi, which corresponds to about 1% of the intestinal cell population (Sternini et al., 2008; Lee and Mezoff, 2021). They play a sensor role by detecting change in the luminal environment such as the presence of bacterial metabolites leading to the production and secretion of peptide hormones to regulate intestinal motility (by acting on the nervous system) or to regulate the metabolism and absorption of nutrients (Beutler et al., 2017).

### Tuft Cells

Tuft cells are secretory and chemosensory cells. The name “tuft” refers to the brush-like microvilli projecting from the cells observed by scanning electron microscopy. They contain abundant vesicles at the apical side that constitute a tubulovesicular system. They act as danger sensors and activate the production and secretion of mediators such as IL-33, TSLP and IL-25 and thus play a role in the initiation of type 2 immune responses to fight parasitic helminths (Gerbe and Jay, 2016).

### M Cells

M cells represent intestinal cells found in a small proportion of follicular-associated epithelium covering mucosal lymphoid tissues, such as large aggregates of B lymphocyte follicles called colonic patches, caecal patches or Peyer’s patches (PPs) in the small intestine. Their cell structure differs from the other intestinal cells. Indeed, microvilli characteristics of enterocytes are missing at the apical surface of M cells, resulting in a « microfold » morphology, hence the name of microfold or M cells (Dillon and Lo, 2019). In addition, the mucus layer that covers other epithelial cells is absent from the apical surface of M cells (Frey et al., 1996). At the basolateral side, the M cells have a pocket containing dendritic cells, macrophages, and T and B cells allowing the initiation of an appropriate mucosal immune response (Neutra et al., 2001). A direct interaction of the B cells with M cells is an essential step required for the acquisition of M cell function (Hsieh et al., 2010). These features promote the role of the M cells in the capture of luminal particles and antigens, the transport through the epithelium and the release to submucosal tissues (Tam et al., 2008). This trait of M cells provides occasions for enteropathogens to promote cellular invasion using this transcytotic pathway (Kraehenbuhl and Neutra, 2000). M cell-like cells have also been described at the tips of intestinal villi and are thus called “villous M cells” (Dillon and Lo, 2019). They show morphological similarity to PP M cells with the characteristic absence of apical microvilli and they can be stained by the lectin Ulex Europaeus Agglutinin-1 which binds to fucosylated molecules. Their origin would come from a trans-differentiation rather than from crypt stem cells like other epithelial cells. The cellular signals or cytokines that are responsible for this trans-differentiation are still undefined. As classical M cells, they presumably provide an ability to capture luminal microparticles and respond to microbial invasion but their location at the tips of the villi suggest a short life span.

## Barrier Function and Regulation of the Intestinal Epithelium

The single layer of the intestinal epithelium represents a barrier between luminal antigens and the underlying immune system. Intestinal passage occurs *via* para- or trans-cellular permeability. Paracellular permeability is a passive transport allowing passage of small molecules and ions and is dependent on the highly regulated network of tight and adherens junctions and desmosomes (Galipeau and Verdu, 2016). Transcellular permeability is an active transport occurring for large molecules, antigens and bacteria and involves endocytosis and receptor-mediated transport (Ménard et al., 2010). Intestinal permeability is highly regulated and can be influenced by many factors including stress, food, inflammation, proteases, etc.

Proteases are produced by all cell types and exert intra- and/or extra-cellular functions. They can affect intestinal permeability directly by their proteolytic action on tight junctions and extracellular matrix proteins and indirectly through activation of proteinase-(in)activated receptors and mediators (Giuffrida et al., 2014; Marshall et al., 2017). Proteolysis represents an effective posttranslational and irreversible regulatory mechanism for modifying the biological activity of proteins and for protein degradation. Therefore, protease activity is tightly regulated by (1) transcription, (2) (narrow) substrate specificity, (3) expression as inactive pro-form that needs to be cleaved for activation (zymogen), (4) compartmentalization, and (5) endogenous inhibition of protease activity (Parks et al., 2004; Marshall et al., 2017). Based on the structure of the active site, which determines the chemical mechanism for the hydrolysis of the peptide bonds, mammalian proteases are classified as serine, cysteine, aspartic, metallo- and threonine proteases. Proteases can be secreted, membrane-bound or remain intracellular.

The gastrointestinal tract is exposed to high levels of endogenous and exogenous proteases, both in the lumen and in the mucosa. Dysregulation of the protease/protease inhibitor balance in the gut contributes to epithelial damage and increased permeability. Excessive proteolysis leads to direct cleavage of intercellular junction proteins, or to the opening of the junction proteins *via* activation of protease-activated receptors. In addition, proteases regulate the activity and availability of cytokines and growth factors, which are also known modulators of intestinal permeability (Gitter et al., 2000).

## *Salmonella* Typhimurium Infection Models

Various models have been used to study STm-host interactions especially at the epithelial interface. Studies based on intestinal epithelial organoid models are beginning to extend our knowledge of STm interaction with several intestinal epithelial cell types. The use of this approach to investigate STm infection will be discussed below as potential perspectives. *In vitro* models of intestinal epithelial cell monolayers have been largely used to described STm interaction with IECs but for most of them, their *in vivo* relevance still needs to be demonstrated. To study the interplay between STm with the intestinal epithelium and more particularly with each cell type in their environment, *in vivo* infections are still the best model available. Among *in vivo* STm infection models, mice are attractive models to better understand the mechanism of STm pathogenesis. In addition, mice can be genetically manipulated and are easy to work with. However, depending on mouse background, mouse can be considered as “resistant” (Santos et al., 2001), or as “susceptible” models (Tsolis et al., 1999). Indeed, susceptible mouse such as C57BL/6 or BALB/c carries mutation in macrophage-encoded *nramp1* (natural resistance-associated macrophage 1) or also named *Slc11a1*, which allows to transport iron out of STm-containing phagosomes, leading to the inhibition STm metalloenzymes necessary for bacterial survival. Thus, susceptible mice infected with STm develop a typhoid like disease mimicking human infection with *S.* Typhi. At the opposite, resistant mice such as CBA/J or 129×1/SvJ expressing a functional Nramp1 do not show symptoms and develop an asymptomatic carriage (Lawley et al., 2008). Pre-treatment of susceptible adult mice with streptomycin and subsequent infection with STm results in an intestinal inflammatory response mimicking gastroenteritis and no longer typhoid fever. This phenotype is explained by reducing intestinal microbiota consequently allowing increased intestinal colonization of STm and thus provides a STm colitis model (Barthel et al., 2003). Another model is the infection of neonatal mice (Zhang et al., 2014a). This model presents the advantage of rapid STm entry into the intestinal epithelium and is characterized by the presence of intraepithelial microcolonies, allowing colonization of both small and large intestine. However, the neonate intestinal epithelium is largely immature and different from the adult epithelium (Renz and Skevaki, 2021).

## Preferential Cell Type Targeted for *Salmonella* Typhimurium Entrance

The intestinal epithelial barrier is the first line of defense between STm and the host. In this section we address the cell type-dependent interaction of STm with the intestinal epithelium and question the relevance of *in vitro* observed mechanisms. STm has the possibility to interact with each cell type of the intestinal epithelium and to use several routes of transports, to escape epithelial defence mechanisms, colonize the epithelium and access to *lamina propria*.

### Enterocytes

Molecular details of STm invasion mechanisms were mainly described in *in vitro* intestinal epithelial cell lines (enterocytes; Han et al., 2021; Aguirre Garcia et al., 2022).

**Figure 2:**
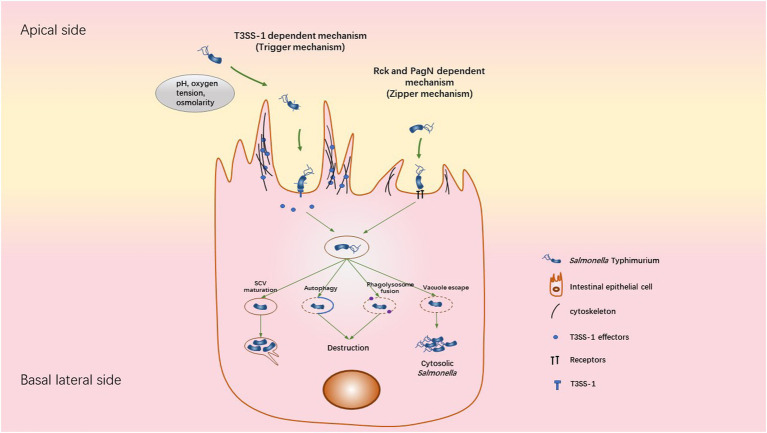
Mechanisms used by STm to invade, survive and replicate inside cell. STm can invade cells using two different ways: the trigger and zipper mechanism. The Trigger mechanism requires the T3SS-1 to directly translocate bacterial effectors into host cells, leading to a massive reorganization of the actin cytoskeleton. Following the invasion, STm is contained in the SCV, where it can replicate. This bacterial replication is accompanied by Sif formation from the SCV, allowing the transport of nutrients to SCV. When the SCV is damaged, the fusion of SCV-Lysosome or the autophagy response could destroy the bacteria. A subset of bacteria is able to escape the SCV into the cytosol. The cytosolic niche is associated with *Salmonella* hyper-replication. The zipper mechanism requires the direct interaction of *Salmonella* outer membrane proteins (Rck or PagN) with a specific receptor (EGFR for Rck or proteoglycan/β1 integrin for PagN) on the membrane surface of the target cell, leading to actin polymerization, weak membrane rearrangement and internalization of the bacteria inside a vacuole. However, the intracellular behavior of *Salmonella* following this invasion process remains unclear.

STm internalization depends on several invasion factors. Until now, the type three secretion system called T3SS-1, two invasin proteins called Rck (Resistance to complement killing) and PagN (PhoP activated gene N) have been identified. The T3SS-1, encoded by the *Salmonella* pathogenicity island 1 (SPI1), allows the injection of bacterial effectors directly into the target cell, characteristic of a “trigger entry mechanism” (Pati et al., 2013). This leads to stimulate Rho GTPase signaling pathways, inducing a massive actin polymerization and membrane remodeling allowing bacterial entry (Figure 2). The second invasion mechanism used by STm is a “zipper mechanism,” which requires a host membrane receptor. The interaction of the outer membrane proteins Rck and PagN with EGFR and proteoglycans/β1 integrin respectively, activates signaling pathways, involving GTPases and actin polymerization (Lambert and Smith, 2008; Hume et al., 2017; Mambu et al., 2017; Barilleau et al., 2021). This mechanism is accompanied by a weaker remodeling of the membrane which is characteristic of a “zipper mechanism” (Figure 2; Rosselin et al., 2010; Barilleau et al., 2021). Furthermore, studies using a STm mutant strain, which does not express Rck, PagN and a functional T3SS-1, reveal the existence of hitherto unidentified invasion factors causing weak or massive membrane rearrangements at distinct entry sites (Rosselin et al., 2011; Roche et al., 2018). These entry mechanisms are more specifically reviewed in (Fattinger et al., 2021c).

Following bacterial invasion, STm is contained within a membrane-bound vacuole called SCV (*Salmonella*-containing vacuole). The SCV relocates to the perinuclear area allowing *Salmonella* to interfere with the host endocytic pathway towards the Golgi apparatus (Figure 2; LaRock et al., 2015; Tuli and Sharma, 2019). This SCV includes an acidified pH environment and acquires membrane markers of endosomal vesicles. The conditions inside the SCV allow the expression by STm of another type 3 secretion system (T3SS-2) encoded by the SPI2. This allows the injection of effectors across the SCV membrane into the host cytosol (Vishwakarma et al., 2014). Briefly, this leads to the manipulation of the host cell vesicle transport to acquire late endosomal markers such as Lamp1 and Rab7. In addition, T3SS-1 effectors (SopB, SopE and SopA) are required for this step of SCV maturation and trafficking. It has been shown that the SCV maturation avoids fusion of the phagosome with the lysosome by a Rab14-dependent mechanism controlled by the T3SS-1 effector SopB. Thus, the environmental conditions inside the SCV are favorable for *Salmonella* proliferation and the bacteria begin to replicate (Kehl et al., 2020). Moreover, this replication phase is accompanied by the formation of tubulovesicular SCV structures called *Salmonella*-induced filaments (Sifs) in addition to actin accumulation underneath the SCV (Figure 2). T3SS-1 (SipA, SopB and SptP) and T3SS-2 (SseF, SseJ, SpvC, SifA, SseG, PipB2, and SpvB) effectors are required for this step of replication/SCV positioning (Kehl et al., 2020).

A subset of STm is able to rupture the SCV and escape into the cytosol of epithelial cells. These cytoplasmic bacteria have the characteristic to hyper-replicate while most STm, which remain within the SCV, replicate at moderate rates (Knodler, 2015). Intriguingly, this cytosolic stage can be considered as a critical step for the bacterial escape into the extracellular environment by damaging the host cell. Dissemination of STm into the environment is thus facilitated, involving apoptosis and pyroptosis of the host cell (Knodler et al., 2010; Watson and Holden, 2010; Flieger et al., 2018). It is currently unclear whether intracellular behavior of STm is affected by the route of cellular internalization.

In streptomycin pre-treated mice infected with STm, the enterocytes constitute the initial target for STm invasion (Müller et al., 2012; Sellin et al., 2014; Zhang et al., 2014a; Fattinger et al., 2021c). But what are the invasion factors required to invade the intestinal epithelial cells *in vivo*? To our knowledge, the molecular details of Rck and PagN mediated invasion mechanism of epithelial cells *in vivo* remains unclear. Until now, a role of Rck in STm fitness has been demonstrated in a resistant murine model (Dyszel et al., 2010). Concerning PagN, a role in bacterial survival and pathological changes in the caecum of streptomycin pre-treated mice has been revealed (Yang et al., 2013). Further investigations are currently in progress to clarify these points. Regarding the T3SS-1, the requirement of T3SS-1 effectors for colonization of the caecum and the small intestine colonization has been shown in the STm colitis and neonate susceptible murine model, respectively (Hapfelmeier et al., 2004; Hapfelmeier and Hardt, 2005). Surprisingly, in streptomycin pre-treated murine caecum, strong membrane rearrangement representative of the *in vitro* T3SS-1 dependent entry mechanism is not observed. STm infection leads to elongation of the microvilli at the luminal surface of enterocytes forming a weak membrane rearrangement. This leads to the bacterial invasion into a vacuolar compartment preferentially near intercellular junctions. However, this mechanism requires the T3SS-1 SipA effector and not the effectors involved in GTPases activation (Fattinger et al., 2020). This study questions on the mechanism used by STm to invade enterocytes *in vivo* according to the STm pathogenesis and the relevance of the data obtained with IEC monolayer cell line.

### M Cells

**Figure 3:**
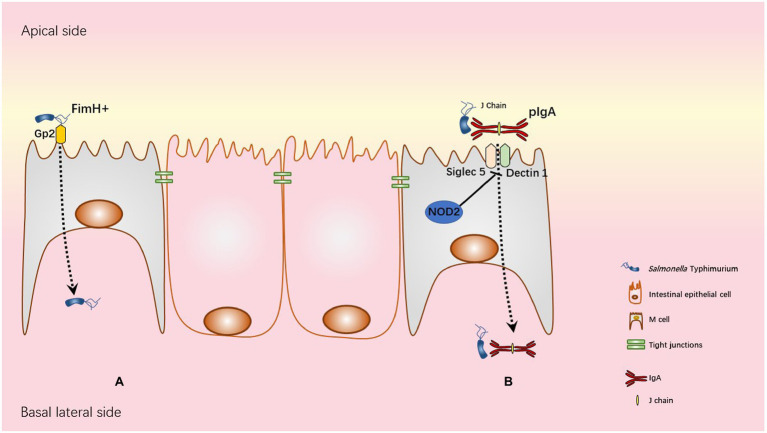
Transcytosis mechanisms used by STm. **(A)** Glycoprotein 2 (GP2), specifically expressed on the apical membrane of M cells recognizes FimH, a component of type I pili on the STm outer membrane. This interaction allows STm transcytosis. **(B)** In physiological condition NOD2 WT is associated with inhibition of both Siglec5 and Dectin1 as represented in the figure. NOD2 polymorphisms R702W, FS1007insC, or R702W/G908R in human Crohn’s disease patients and Nod2 deficiency in mice are associated with overexpression of both Siglec5 and Dectin1 in M cells. Upregulation of Siglec5 and Dectin1 allows retrograde transport of pIgA (polymeric IgA)/STm immune complexes from the apical to basolateral side of M cell.

Using ligated-loop model of susceptible mice, it has been pointed out that STm exploits M cells as a route of invasion and colonize PPs (Jensen et al., 1998). In addition, using *in vivo* mouse and *in vitro* primary rectal epithelial cells infection models, it has been demonstrated that the T3SS-1 SopB effector of STm induces the trans-differentiation of follicular-associated epithelium enterocytes into M cells. Thus, during infection, the number of M cells increases to promote host invasion and colonization (Tahoun et al., 2012). M cell invasion seems to occur through both T3SS-1-dependent and -independent mechanisms. Indeed, STm strains defective for T3SS-1 are still able to invade M cells but less than wild-type STm strain (Clark et al., 1998; Martinez-Argudo and Jepson, 2008). On the other hand, the use of mouse strains lacking organized gut-associated lymphatic tissues and lymph nodes, allowed to demonstrate that mesenteric lymph nodes and PP are dispensable for the colitis induction in streptomycin pre-treated mice (Barthel et al., 2003). The role of M cells as a privileged entry site for STm remains under discussion but recent studies in mice and in humans uncovered two distinct mechanisms of STm entry into M cells, involving IgA/STm/dectin1-Siglec5 and FimH/Glycoprotein 2 (GP2; Figure 3).

Secretory IgA (SIgA) produced by B cells in the lamina propria and delivered into the gut lumen *via* transcytosis through polymeric immunoglobulin receptor (pIgR) is the dominant immunoglobulin at mucosal surfaces and is known to be an important player in defence mechanisms against enteric pathogens (Wijburg et al., 2006; Bioley et al., 2017). However, retro/reverse-transcytosis (from the lumen to lamina propria) of IgA/pathogens immune complexes can act as a Trojan horse and worsen infection as observed in human Crohn’s disease (Rochereau et al., 2021). Crohn’s disease is an inflammatory bowel disease with multiple origins, i.e., genetic and environmental. Interestingly, STm-mediated human gastroenteritis increases the risk to develop Crohn’s disease (Schultz et al., 2017). Among genetic factors associated with Crohn’s disease, NOD2 mutation affects intestinal barrier functions resulting in increase of intestinal permeability (Al Nabhani et al., 2016). Interestingly, NOD2 mutation (loss of function) increases expression of Dectin1 and Siglec5 on M cells leading to an increased entry of IgA/STm immune complexes (Rochereau et al., 2021) in human PP and susceptible mouse models. Another uptake mechanism involving FimH and GP2 has been described in M cells. GP2 was one of the M cell-specific molecules identified in human and mice (Hase et al., 2009). GP2 recognizes FimH, a major component of the type 1 pilus on the outer membrane of a subset of gram-negative enterobacilli like *Salmonella enterica* (Hase et al., 2009). GP2-FimH interaction is necessary for efficient uptake of FimH+ STm by M cells and subsequent bacteria-specific mucosal immune responses as demonstrated in “susceptible” C57BL/6 mice.

### Goblet Cells

To avoid its elimination from the intestine, STm has to adhere to and penetrate the protective mucosal layer and there is evidence showing STm binding to mucus through flagellar motility and chemotaxis in streptomycin pre-treated mice (Nevola et al., 1987; McCormick et al., 1988; Vimal et al., 2000; Stecher et al., 2004). More particularly, it has been pointed out that STm expresses Std fimbriae in gastrointestinal tract of streptomycin pre-treated mice, allowing the bacterial binding to fucosylated structures in the mucus (Chessa et al., 2009; Suwandi et al., 2019). Mucus represents a physical and chemical (presence of anti-microbial peptides (AMP) and IgA) barrier preventing STm’s direct access to intestinal epithelial cells. Indeed, using explant of susceptible C57BL/6 mice pre-treated with streptomycin, Furter et al. demonstrated that mucus is necessary to prevent STm adherence to the intestinal epithelium (Furter et al., 2019). Using live microscopy, the authors showed that STm has direct access to IECs in the caecum where the epithelium between crypts is unshielded. However, this direct access is prevented in the colon by a dense inner mucus layer covering the epithelium and/or luminal content. This study provides an explanation for the caecum being a primary site of STm infection in the streptomycin-treated mouse model (Furter et al., 2019). Until now, there is no evidence of *Salmonella* invasion into goblet cells. However, the lack of continuous mucus layer in the caecum segment may explain why in the streptomycin mouse model, the caecum is the highly permissive and the intestinal segment preferentially infected by STm.

Goblet cells are responsible for goblet Cell Associated Antigen Passages known to participate in oral tolerance by facilitating antigen passage and recognition by immune cells (Knoop and Newberry, 2018). In addition, tight junctions associated with goblet cells display structural variability such as paracellular space, which is not found between villus absorptive cell tight junctions in rat ileal mucosa. This suggests focal sites of high permeability between villus goblet cells, contributing to the relatively low resistance to ionic flow (Madara and Trier, 1982). This is coherent with the observations of Fattinger et al. showing that STm are enriched in the neighborhood of goblet cells in the caecum of mice infected with STm (Fattinger et al., 2020b). Thus, it is not excluded that STm takes advantage of the paracellular spaces next to goblet cells to translocate into the intestinal epithelium.

### Stem Cells

In streptomycin pre-treated mice, the presence of mucus in caecal crypts prevents the attachment of STm, whereas the upper parts of the crypt epithelium are devoid of mucus and thus allows attachment of STm (Furter et al., 2019). In 1970s, using scanning electron microscopy, bacteria were already visualized attaching to mouse intestinal crypts *via* long filamentous extensions (Nelson and Mata, 1970; Savage and Blumershine, 1974; Palestrant et al., 2004; Donaldson et al., 2016). Thanks to the development of fixation methods preserving microbial biofilm, microbial populations that are based within intestinal crypt were visualized and subsequently identified using 16S rRNA sequencing (Peck et al., 2017). Recent studies suggest the caecum and the colon as intestinal segments containing crypt-based microbiota, which is coherent with the microbial density found within the intestine (Swidsinski et al., 2005; Pédron et al., 2012). Not surprisingly, enteropathogens have developed strategies to gain access to intestinal crypts. For example, this is the case for *C. jejuni* and *V. cholerae*, which were found associated within crypts in the caecum and small intestine of C57BL/6 mice, respectively (Yang and Ottemann, 2019). In STm-infected intestine, STm were found within crypts and an alteration of the expression of *lgr5+* (Leu-rich repeat containing G protein-coupled receptor 5) gene, a stem cell marker, localization and number of stem cells within the crypts were also observed in susceptible and resistant mice pre-treated or not with streptomycin (Liu et al., 2010; Lu et al., 2012; Martinez Rodriguez et al., 2012; Rossi et al., 2012; Santos et al., 2016). However, STm invasion into stem cells has not been reported until now, probably due to the absence of antibodies available to specifically identify stem cells.

### Other Cell Types

Paneth cells belong to the secretory lineage but they have also been identified as facilitating entry for luminal antigens. In the small intestine of STm-infected susceptible mice, secretory cells hyperplasia is observed. More particularly, an increase of the number of goblet, paneth, and tuft cells per crypt has been demonstrated (Lu et al., 2020). STm invasion into tuft cells, paneth cells or enteroendocrine cells has not been reported.

## Defence Responses of the Intestinal Epithelium to STm Infection

Following STm infection, the intestinal epithelium develops strategies to fight back and clear the pathogen. In the following paragraphs, the global responses of the intestine, following by the specific responses of the differentiated secretory epithelial cells, will be described.

### Diarrhea

In mice with STm-induced colitis, SCV internalized STm secretes a heat-labile enterotoxin responsible for efflux of water and electrolytes into the intestinal lumen aiming to flush the pathogen (Finlay and Falkow, 1988). This diarrheagenic enterotoxin is released into the cytoplasm of invaded cells and into the intestinal lumen. The diarrhea is caused by a higher secretion of chloride ions in the crypt region and decreased absorption of Na^+^ in intestinal villi (Fromm et al., 1974; Khurana et al., 1992). In addition, STm infection increases cell proliferation, affecting absorptive function in caecum and colon of antibiotic pre-treated mouse model, which has also been shown to drive diarrhea and increases fecal water content dependent on the T3SS-1 (Fierer et al., 2012; Marchelletta et al., 2013). Therefore, a therapeutic strategy to re-establish absorptive transport would be beneficial for patients suffering from STm induced diarrhea.

### Epithelial Chemokines and Retinoic Acid Response

Infections with STm induced acute intestinal inflammation in human and animal hosts, as a result of the bacterium invading the mucosa. The intestinal epithelial cells sensors the bacteria and initiate intestinal innate immune responses.

STm attachment to polarized IEC human cells induces basolateral secretion of interleukin-8 (IL-8). In Caco2 human cell lines, IL-8 secretion is induced by STm flagellin recognition by TLR5 and potentiated by activation of NOD2 by muramyl dipeptide (Huang, 2012). Flagellin-induced IL-8 secretion also involved SopE2, a *Salmonella* guanine nucleotide exchange factor (Huang et al., 2004). Interestingly, when STm invades transformed human colonic epithelial cell line, a cholesterol accumulation in SCVs lipid rafts is induced. This activates the PI3K/Akt pathway contributing to dampen IL-8 secretion and reducing ERK signalling pathways (Huang, 2011). Once intracellular, STm therefore favors its colonization by decreasing IL-8 production in human intestine-407 cells by a mechanism involving the translocation of the effector proteins SspH1 and SptP (Haraga and Miller, 2003). Neutrophil recruitment is generally attributed to IL-8 secretion. But transmigration of neutrophils toward the apical side of epithelial cells is also dependent on apical release of pathogen-elicited epithelial chemoattractant bioactivity by IEC (T84; McCormick et al., 1993, 1998). IL-8 and pathogen-elicited epithelial chemoattractant secretions induced by STm were suggested to involve distinct signalling pathways (Gewirtz et al., 1999). STm also triggers CCL20 basolateral secretion by IECs and subsequent recruitment of dendritic cells initiating adaptive immune response in the gut (Sierro et al., 2001; Caco-2 and T84 cells). This process is dependent on flagellin.

Furthermore, STm interaction with IEC modulates intestinal immune response including AMP, IL-17 and IgA secretion by metabolizing vitamin A into retinoic acid resulting in control or exacerbation of infections (Peterson and Artis, 2014). In susceptible mice depleted in IEC for (Rdh7) involved in vitamin A metabolism in retinoic acid, the STm load in feces and systemic dissemination are significantly reduced after oral infection. This effect is reversed by exogenous retinoic acid, showing a deleterious role of retinoic acid in STm infection (Grizotte-Lake et al., 2018). Retinoic acid production *in vivo* by IEC of streptomycin pre-treated mouse leads to IL-22 expression in immune cells then AMP secretion and microbiota dysbiosis favoring STm colonization (Behnsen et al., 2014; Miki et al., 2017; Grizotte-Lake et al., 2018). Recently, the same group described opposite results on a streptomycin pre-treated mouse model expressing dominant negative of retinoic acid receptor in their IEC. Susceptibility to luminal and systemic colonization by STm is higher in those mice due to a weaker induction of protective IL-18 by epithelial cells. These two studies highlight the autocrine and paracrine functions of IEC-produced retinoic acid during STm infection (Iyer et al., 2020).

The canonical inflammasome (Caspase 1) represents an important pathway for IL-18 release and restriction of STm replication in the mouse caecum *in vivo*. *In vitro* studies comparing transformed and untransformed IEC (enteroids derived monolayers) derived from mice and human showed that Caspase-1 is important for restricting intracellular STm replication and IL-18 secretion in mouse IECs but is dispensable in human IECs. This study highlights species differences. However, in both transformed and untransformed human IECs, Caspase-4 is mandatory for restriction of intracellular STm and production of IL-18. Importantly, cytosolic replication is lower in untransformed cells compared to transformed cells in both species highlighting bias in *in vitro* cells experiments (Holly et al., 2020).

### Epithelial Host Proteases

STm infection of the epithelium leads to changes of host protease expression by direct (infection of the cell) and indirect mechanisms (induction by cytokines/chemokines). Host cell proteases are involved in every step of the infection process from invasion, intracellular trafficking, survival and exit of the cell. Thus, proteases play a key role in the defence against invading pathogens by attacking STm in the different segments of the intestine using intracellular and extracellular functions. However, STm also manipulates proteases to modulate host tissues for invasion or evasion from the host immune system (Marshall et al., 2017).

#### Intracellular Functions

In intestinal Henle-407 cell line, it has been demonstrated that STm utilizes the proteasome-dependent protein degradation pathway to temporally regulate virulence effector functions. The secretion and translocation domain determines the kinetics of degradation leading to a short half-life of SopE, which regulates cytoskeleton rearrangement for invasion, whereas SptP, which is needed to reverse these effects afterward, is more stable in the host cell cytoplasm (Kubori and Galán, 2003).

In addition, the process of autophagy targets cellular macromolecules or cytosolic pathogens (xenophagy) for proteolytic degradation in newly formed vesicles (autophagosomes) which fuses with lysosomes to form autophagolysosomes. In autophagolysosomes, degradation is catalyzed by various cathepsins. Furthermore, autophagy is negatively regulated by cysteine proteases, such as caspases, that can cleave autophagy-related proteins (Kaminskyy and Zhivotovsky, 2012). In human intestinal cell line, it has been shown that the autophagy process acts transiently at early time points of STm infection (Knodler, 2015). Autophagy is initiated by damaged SCVs or by STm that escaped into the cytoplasm (Wu et al., 2020). A few studies found that autophagy can also facilitate cytosolic STm replication (Wu et al., 2020). STm inhibits autophagy by several mechanisms (Knodler and Celli 2011; Sharma et al., 2018; Wu et al., 2020), but the direct impact of STm on the regulation of these cathepsins has not yet been investigated.

#### Extracellular Functions

Besides intracellular functions, some cathepsins are also secreted into the gastrointestinal tract and play a role in homeostasis (extracellular matrix remodeling, barrier function) or inflammation (cytokine/chemokine processing; Vidak et al., 2019). Colonic mucus samples containing cathepsin K from wild type mice impaired *in vitro* growth of STm (and certain commensals) in contrast to mucus from cathepsin K deficient mice (Sina et al., 2013) suggesting a direct antimicrobial activity of cathepsin K.

Dependent on the context, matrix metalloproteinases (MMPs) can have anti- or pro-inflammatory activity. *In vivo*, STm infections results in transcriptional upregulation of MMPs family genes, *Mmp3*, *Mmp8*, and *Mmp28* in PPs and mesenteric lymph nodes (mLNs) 2 days post-infection (Handley and Miller, 2007) and further *Mmp* genes in the inflamed caecum up to 3 weeks post infection. In this context, protein expression of MMP3, MMP7, MMP8 and MMP10 were detected in epithelial cells in the STm-infected caecum (Ehrhardt et al., 2019). MMP3 deficient mice showed a delayed colonization of PPs, mLNs and spleen (Handley and Miller, 2007).

ECM degradation by MMP-9 impairs human intestinal cell line attachment and wound healing (Castaneda et al., 2005) and MMP-9 leads to disruption of tight junctions in a human intestinal epithelial cell line *in vitro* (Al-Sadi et al., 2021). *Mmp9* is not expressed in normal colonic mucosa, whereas *Mmp2* is constitutively expressed, but both are up-regulated in intestinal cells *in vivo* during STm infection (Castaneda et al., 2005; Garg et al., 2006). MMP-9 deficient mice have reduced inflammation and intestinal tissue damage and were protected against STm induced colitis (Castaneda et al., 2005). Epithelial derived MMP-9 is responsible for the increased susceptibility. In this line, MMP-9 does not affect the immune response to systemic infection with STm (Castaneda et al., 2005). In contrast, MMP-2 deficient mice are highly susceptible to STm-induced colitis (Garg et al., 2006). Epithelial derived MMP-2 protects the epithelial barrier integrity and promotes healing, which is compromised in MMP-2 deficient mice and may lead to their increased susceptibility to STm infection. This opposing effects of the two MMPs on the barrier permeability might be explained by interaction with different tight junction proteins and/or non-matrix substrates; MMP-2 associates with claudin, whereas MMP-9 cleaves occludins (Garg et al., 2006). Furthermore MMP-2 degrades MCP-3, fibroblast growth factor receptor-1, and possibly other chemoattractants, while MMP-9 does not (Garg et al., 2009). MMP9 and MMP2 double-deficient susceptible mice are resistant to STm-induced colitis demonstrating that MMP-9 overrides the protective effect of MMP-2 in mediating inflammation and tissue damage (Garg et al., 2009). In resistant mouse infection model, no change in expression of these MMPs in comparison with uninfected controls was detected indicating that they play a role in acute but not chronic infections (Ehrhardt et al., 2019).

#### Caspase Induced Cell Death

Caspases are intracellular cysteine aspartic proteases involved in the different forms of cell death and activation of the cytokines IL1-β and IL-18 and further inflammatory processes. Distinct caspases are part of a higher protein complex called the inflammasome. Besides the well-studied role of inflammasomes in immune cells like macrophages, their role in immune signalling and restriction of STm growth in epithelial cells becomes more apparent. Caspase activation and inflammasome formation is induced by pathogen sensing *via* pathogen-associated molecular patterns (PAMPs) or stress sensing *via* damage-associated molecular patterns (DAMPs). Dependent on the way of activation, canonical and non-canonical inflammasomes are distinguished. Canonical inflammasomes contain Caspase 1, and they are activated *via* sensing of pathogens *via* cytosolic pattern recognition receptors (PRR). In contrast, non-canonical inflammasomes composed of human Caspase 4/5 or the mouse homologue Caspase 11 directly bind to cytosolic LPS. STm activates canonical inflammasomes (e.g., NAIP/NLRC4, NLRP3, AIM2) by, e.g., flagella and T3SS-1 components. Furthermore, using both human intestinal cell line and enteroids models derived from mouse and human, it has been demonstrated that the SCV can be damaged or STm can escape the SCV and (hyper-)replicate in the cytoplasm which is sensed and restricted by Caspase 4/11 (Holly et al., 2020). It is suggested that in murine intestinal epithelial cells the NLRC4 inflammasome is induced early followed by Caspase 11 activation at later time points (Fattinger et al., 2021a,b). Engagement of different inflammasomes might be dependent on the cell type, the differentiation status of the cell, and the species (Holly et al., 2020; Fattinger et al., 2021c). Activated caspases process pro-inflammatory pro-IL-1β and/or pro-IL-18, and Gasdermin-D initiating pyroptosis, an inflammatory type of cell death, and epithelial cell extrusion. Cleaved Gasdermin-D forms pores in the plasma membrane leading to pyroptosis and secretion of the mature cytokines. Expulsion of pyroptotic epithelial cells into the intestinal tract of streptomycin pre-treated mice limits bacterial burden in the tissue and dissemination to the mLNs (Sellin et al., 2014; Hausmann et al., 2020; Fattinger et al., 2021a). In case of STm susceptible mouse infection, escape of the cytosolic (invasion primed) bacteria into the gut lumen by pyroptotic cells provides a potential mechanism of dissemination (Knodler et al., 2010).

AIM2 recognizes dsDNA and restricts STm infection by inducing tight junction protein expression and promoting the proliferation/apoptosis homeostasis thereby strengthening the epithelial barrier (shown in susceptible mice and in Caco2 cells; Hu et al., 2016). For details on inflammasome activation and their role in the host defence to STm, see the recent comprehensive reviews (Clare, 2021; Fattinger et al., 2021b).

Activation of multiple other caspases, which are involved in cell death during STm infection, lead to mixed cell death signalling resulting in PANoptosis (Fattinger et al., 2021b). Epithelial Caspase-8 restricts STm murine intestinal colonization and protects the intestinal barrier by shifting the cell death pathways from necroptosis to apoptosis in a streptomycin pre-treated mouse and murine intestinal organoid model (Hefele et al., 2018). Therefore C57Bl/6 mice deficient in epithelial Caspase-8 are highly susceptible to STm infection (Hefele et al., 2018; Stolzer et al., 2022). Besides apoptosis, Caspase-3 also exerts other functions. In infected intestinal epithelial cells, SipA is involved in cell invasion and activates Caspase-3 and promotes its apical secretion. Caspase-3 subsequently cleaves SipA into two functional subunits promoting transepithelial migration of neutrophils in susceptible mouse model (Srikanth et al., 2010). Mutation of the Caspase-3 cleavage site in SipA or infection of Caspase-3 deficient C57Bl/6 mice leads to a reduced caecal inflammation. Caspase-3 cleavage site(s) are found in several STm virulence proteins and inactivation by mutation reduces their pathogenicity of SopA (Srikanth et al., 2010) and SifA (Patel et al., 2019).

#### Mucus Layer

The mucus layer plays an important role in defence against STm. Indeed, MMP-9 negatively regulates goblet cell differentiation and consequently secretion of mucin-2 (MUC-2), which is a major component of the protective mucus gel layer on the intestinal epithelium. MMP-9 overexpression (e.g., in infection) or silencing in goblet cells, decreases or increases MUC-2 expression and thus increased or decreased STm adherence, respectively (Garg et al., 2007).

It has been described that upon activation of EGFR, goblet cells increase mucin production (Nadel, 2001). This could lead to the formation of a protective layer of mucus by limiting bacterial access to the epithelium as STm expresses Rck which is able to interact with EGFR expressed on epithelial cell line (Wiedemann et al., 2016). However, the impact of the interaction of Rck with EGFR on mucus production has not been clarified.

#### Antimicrobial Peptides

Antimicrobial peptides (AMPs), including defensins and cathelicidins, are innate small molecules playing a key role in intestinal host defence. They are secreted by different cell types and among intestinal epithelial cells, but mainly by paneth cells. After STm infection of streptomycin-treated-mice, single cell transcriptomic analysis of epithelial cells revealed an increased number of mature paneth cells. In addition, an increase of AMPs gene expression such as DEFA23, DEFA24 and Ang4, and of Pentraxin2 was observed in paneth cells (Haber et al., 2017). In another mouse model of infection with FvB mice (susceptible model), STm was shown to increase the number of paneth cells but inhibit Cryptdin (α-defensin) and lysozyme expression as soon as 1 day after infection by a mechanism involving T3SS-1 (Salzman et al., 2003a; Martinez Rodriguez et al., 2012). This suggests an escape mechanism induced by the bacteria. As STm is not directly in contact with paneth cells, it is conceivable that infected enterocytes release specific mediators which would signal to paneth cells. Another explanation is that STm infection triggers intestinal progenitor cell proliferation and the initiation of a paneth cell differentiation program resulting in increased paneth cell numbers with decreased granule content (Martinez Rodriguez et al., 2012; Haber et al., 2017). Transcriptomic analysis of AMPs have to be handled carefully as AMP are highly regulated after translation. α-defensins are produced as pro-peptides and their activation depends on MMP-7. MMP7^−/−^ C57Bl/6 deficient mice are more susceptible to oral challenge with STm, highlighting the role of paneth cells AMP in STm infection (Wilson et al., 1999). Transgenic mice expressing human α-defensin-5 (DEFA5) in paneth cells, in addition to the mouse AMP repertoire, showed increased resistance to oral STm challenge (Salzman et al., 2003b). Even though direct bactericidal effects of HD5 and α-def have been demonstrated on STm *in vitro* (Selsted et al., 1992) an indirect role of AMP on STm *via* modulation of host microbiota needs to be taken into account. Indeed, AMP can shape intestinal microbiota without affecting the total number of bacteria (Salzman et al., 2010). Both mouse models, either complemented with human DEFA5 or deficient for MMP7 (deficient of α-defensins) are characterized by microbiota dysbiosis. Susceptibility to STm can therefore also be a consequence of an altered resident microflora. This was also suggested with AMP such as C-Type lectin Reg3 β/γ that are over-expressed during STm infection of streptomycin-pretreated mice but have no effect in STm-killing. Reg3 β/γ can kill diverse commensal gut bacteria which could favor growth advantage to STm over microbiota (Godinez et al., 2009; Stelter et al., 2011). The AMP repertoire is also dependent on the mouse genetic background (Gulati et al., 2012) and could explain that - in addition to *Nramp1/Slc11a1* status - some mouse strains are more resistant to STm infection. Indeed, susceptible C57BL/6 mice complemented with Nramp1 remained more susceptible than resistant 129Sv mice (Brown et al., 2013). Goblet cells can also secrete some antimicrobial molecules such as cathepsin K into the mucus and its antimicrobial property was demonstrated *in vitro* against STm (Sina et al., 2013).

Independently of their environment but according to their virulence, STm are more or less sensitive to AMP. *In vitro* killing assays demonstrated that neither cryptdin 1 nor cryptdin 2 were able to kill mouse-virulent, phoP^+^ STm 14028S, despite their ability to kill avirulent, isogenic, phoP congener, STm 7953S (Eisenhauer et al., 1992). These data are in accordance with previous work demonstrating that the sensitivity of STm to defensins and its virulence for mice are associated and dependent on the PhoP-PhoQ regulon which controls the expression of several genes, including *pagC* (Fields et al., 1989; Groisman et al., 1989; Miller et al., 1989).

STm was shown to inhibit paneth cell AMP expression as soon as 1 day after susceptible mouse infection. This effect is mediated by STm T3SS-1 but does not involve paneth cell apoptosis (Salzman et al., 2003a; Martinez Rodriguez et al., 2012). Cryptdin and lysozyme are the two AMPs for which mRNA expression was reduced after infection suggesting an escape mechanism induced by the bacteria. As STm is not directly in contact with paneth cells, it is conceivable that infected enterocytes release specific mediators which would signal to paneth cells. Another explanation is that STm infection triggers intestinal progenitor cell proliferation and the initiation of a paneth cell differentiation program resulting in increased paneth cell numbers with decreased granule content (Martinez Rodriguez et al., 2012; Haber et al., 2017).

#### Hormones

Hormones are produced by enteroendocrine cells in response to several stimuli such as nutrients and microbial metabolites to mediate digestion and orchestrate intestinal inflammation. Daly et al. have demonstrated that in STC-1, mouse enteroendocrine cell line, heat-inactivated STm induced cholecystokinin, a satiety hormone, producing contraction of the gall bladder and concomitant release of bile. Even though this mechanism has to be confirmed *in vivo*, enteroendocrine cell seems to participate in the expulsion of STm (Daly et al., 2020).

## Relevance of Intestinal Epithelial Organoids to Decipher *Salmonella*-Epithelial Cell Crosstalk

*In vitro* analysis of *Salmonella* interaction with the primary intestinal epithelium, has been hampered by the absence of appropriate *in vitro* culture. Since 2009, starting with a landmark study by the Clevers’ lab (Sato et al., 2009) progress in the research on intestinal epithelial stem cells has allowed the emergence of 3-dimensional (3D) multicellular structures called “intestinal epithelial organoids,” representing the micro-anatomy of a functional intestinal epithelium *in vitro*. Intestinal organoids are derived from stem cells with self-renewal and pluripotency properties, allowing them to generate all the cells reconstituting the intestinal lineage *in vitro*. Three types of stem cells are commonly used to derive intestinal organoids: (i) induced pluripotent stem cells, which are somatic cells genetically reprogrammed to make them pluripotent; (ii) embryonic stem cells; (iii) adult stem or progenitor cells isolated from intestinal tissues.

These primary epithelial cells have physical, cellular, molecular and genetic characteristics similar or even identical to those of the tissues *in vivo*. Specific culture conditions are required such as the use of Matrigel^™^ (composed of Type IV collagen, laminin and fibronectin) in order to mimic the physiological microenvironment of the extracellular matrix *in vivo* (Sato et al., 2009), as well as several growth and differentiation factors, playing an essential role in the regulation of epithelial homeostasis (Barker, 2014). During cellular differentiation, this intestinal epithelium becomes polarized and apoptotic cells appear in the central lumen. The polarity of the cells is then identical to that of the cells of the intestine, with an apical pole in the center of the organoid and a basal pole towards the outside of the organoid. The organoids mature and form buds, representing the intestinal crypt. The maintenance of homeostasis and intestinal integrity are based on the existence of an intercellular communication network within the epithelium itself. However, the organization of the 3D intestinal organoid limits the access of pathogens from outside the organoids to the epithelial surface on the side of the intestinal lumen (Yin et al., 2016). To bypass this problem, it is possible (i) to use a microinjection system to directly deliver pathogens into the 3D organoid containing a luminal intestinal structure, thus allowing the access of the apical side. However, it is a time consuming and arduous technique, questioning about reproducibility as the injected volume may vary between each organoid and also leaks may occur at the injection site when withdrawing the needle from the organoid; (ii) “to reverse” the polarity of organoid by removing the extracellular matrix and maintaining the organoid in suspension (Co et al., 2019). However, this technique fails to reverse 100% of the organoids, leading to a heterogeneous phenotype of the organoid population in culture; (iii) to grow organoids as monolayers on Transwell^®^ filter membranes, allowing electrophysiological and permeability studies using Ussing chambers to investigate different functions of the intestinal epithelium. However, generating and maintaining an oxygen gradient across the epithelial monolayer stays a challenging perspective; (iv) to infect dissociated organoid cells and then seed them in Matrigel^™^ to reform organoids. This infection model allows the analysis of the impact of pathogens on the proliferative progenitor and stem cells and thus on the intestinal renewal, development and differentiation. The disadvantage of this technique is the probable loss of cell polarity once the organoid cells are dissociated, which means that the pathogen exposure is not representative of what is going on *in vivo*.

**Figure 4:**
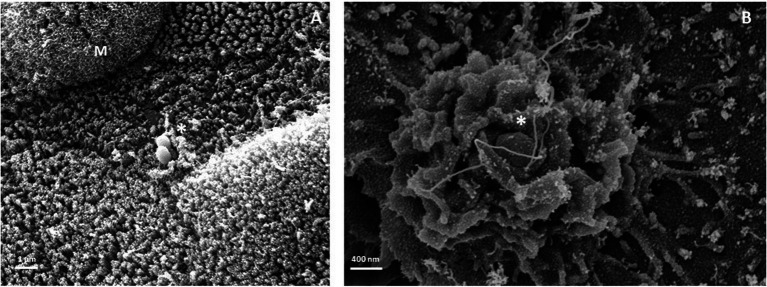
Membrane structures during STm invasion at the apical site of human monolayer organoid derived from colon. Representative image obtained by scanning electron microscopy of discreet **(A)** and strong **(B)** membrane rearrangements. Star indicates STm membrane rearrangement at the entry site. M, mucus. Scale bar: **(A)** 1 μm **(B)** 400 nm.

Thus, the intestinal organoid model represents an interesting experimental model for investigating the interaction of STm with mouse and human intestinal epithelium and visualizing (i) the invasiveness of pathogens either on the basolateral side or on the apical side of the intestine (ii) morphological changes of primary cells such as proliferation of intestinal stem cells as well as their differentiation; (iii) the pathophysiology of pathogen-epithelial cell interactions during infections; (iv) role of certain cell populations such as paneth cells, for which no cell lines are available. Intestinal organoids derived from mouse and human have recently been used to model STm infection and their culture conditions are compatible with STm infections. Moreover, some studies confirm results obtained in several mouse and human intestinal cell lines. Thus, it is a promising STm infection model to investigate the molecular details of the interaction of STm with each cell type present in the intestinal epithelium and more particularly in human. Indeed, after apical microinjection of STm in human intestinal organoids, STm was able to invade epithelium and residing in SCV (Forbester et al., 2015). Furthermore, basolateral STm infection of intestinal organoid in the extracellular environment the organoid has allowed to visualize bacteria inside buds of organoids derived from ileal mouse intestine (Zhang et al., 2014b) as it was observed in small intestine of STm-infected susceptible mice (Santos et al., 2016). Production of functional AMPs in the lumen of organoids derived from susceptible mouse small intestines allow to restrict growth of STm directly microinjected in organoid lumen (Wilson et al., 2015). Moreover, the authors pointed out a decrease of mRNA expression of Lgr5^+^, a stem cell marker, and disruption of tight junctions. In addition, comparative analysis of gene expression and microscopy has shown similarities between responses obtained *in vivo* in susceptible mice and *in vitro* in murine intestinal organoids following STm infection of dissociated organoids. For example, secretory cell hyperplasia (goblet, paneth, and tuft cells), mucus, and AMP production such as Reg3γ and DEFA1 were induced in response to STm infection (Lu et al., 2020). In addition, similar results indicating an inhibition of the cell turnover at the intestinal epithelium and intestinal epithelial secretion induced by STm infection in susceptible mice (Santos et al., 2016) were also observed using human intestinal organoids microinjected with STm to model the bacterial infection at the apical side (Abuaita et al., 2021). Interestingly, organoids from juvenile inflammatory bowel patient failed to control STm growth and highlight the role of IL22 and IL10RB gene in antimicrobial defense against STm (Forbester et al., 2018). Furthermore, as shown in Figure 4, “discreet” and “strong” invasion structures at the apical entry site of STm are also observed near mucus in human organoid monolayers derived from colon (Figure 4). Recent studies on human intestinal organoids showed that STm induced pro-inflammatory gene expression and downregulation of genes associated with cell cycle and DNA repair affecting the host proliferation machinery (Lawrence et al., 2021). Besides the 3D organoid model, 2D organoid monolayer model allow imaging of infection and interaction between IECs and STm as well as the importance of M cells providing an interesting tool to identify STm interaction with specific IEC (Nickerson et al., 2021; van Rijn et al., 2022).

Taken together, these studies allow to conclude that the observed effects on the intestinal epithelium were similar to those obtained in murine colitis infections and intestinal cell lines. Thus, it has emerged that the organoids derived from intestine is an appropriate system to model STm interaction with epithelial intestinal cells, even when the STm infection does not only take place at the apical side (Verma et al., 2020; Aguirre Garcia et al., 2022). This system makes possible to respond to a societal demand aimed at reducing the use of animals for research and is part of the 3Rs approach (Replacement, Reduction and Refinement).

However, the advantage of organoid model being restrictive to intestinal epithelium is also its limitation as it does not reflect the complex interaction with microbiota, immune system, enteric nervous system, etc. Recent advances have shown that it is now possible to further modify the intestinal organoid culture to mimic the *in vivo* environment. The establishment of microfluidics system called organ-on-a-chip allows the addition of environmental element such as microbiota, immune system cells, enteric nervous system. More recently, to mimic the digestive system, intestinal organoids were connected to pancreas and liver organoids in order to investigate the communication between these organs (Koike et al., 2019). However, it is important to understand the challenge of supplementing these elements to a system already quite complex. Each system has its advantages and disadvantages. The choice of the appropriate system is therefore important and dependent on the scientific question to answer.

## Conclusion

The interaction with the non-phagocytic cells of the intestinal epithelium is a crucial step for STm pathogenesis. Indeed, STm has to invade the intestinal barrier and to overcome the secretory defence mechanisms of the host to establish infection. In this review, we tried to highlight the current knowledge regarding the molecular dialogue between STm and the murine and human IECs. Most of the molecular mechanisms described of STm interaction with IECs have been studied in intestinal epithelial cell lines. In this review, we aimed to remind that intestinal epithelium is complex and made of various cell types. Available data on *in vivo* experiments mainly in susceptible mice with or without pre-treatment with streptomycin are scarce and inconsequent to provide information regarding specific interaction between STm and different intestinal cells composing the epithelium. We still think that STm entrance in IEC and involvement of specific cells is a crucial step to better understand salmonellosis and propose treatment. Indeed, a clear identification of the epithelial cells interacting with STm for entry and defense will represent of crucial knowledge for development of targeted therapeutic aiming to block STm entrance and/or to enhance the existing defense mechanisms. It will also represent an interesting tool to highlight differences in *Salmonella* interaction with IECs between mice and human that could contribute to provide explanation for discrepancies in diseases manifestation (typhoid fever in human). The use of organoids derived from livestock animals like chickens (STm host) and non-human primate or pig could be generated to draw an overview of STm interaction with IECs of various species concerned by STm infection. We also propose that it will be an interesting tool to better understand serovar evolution and switch in population like those observed in Africa (Feasey et al., 2012; Bornstein et al., 2019). The organoid technology becoming more sophisticated, the method for quantitative gene expression analysis (RNA-seq) has rapidly been adapted to organoid models and more recently at the single cell level. It is a key approach to high-throughput transcriptome profiling to better understand regulation of pathways involved in *Salmonella* pathogenesis (Nickerson et al., 2018; Ross et al., 2021). Furthermore, interaction with IECs might commit the resulting immune response toward STm and as such a better understanding of STm interaction with IECs might contribute to the vaccine strategy. However, questions still remained: where does the initial interaction/invasion of STm occur? What are the intestinal segments and the cell types preferentially targeted by STm? According to the *in vivo* murine model, has STm the same gateway as target? In addition, human and mouse intestine anatomy are different, therefore, is it possible to translate results obtained in mouse to human?

The recent development of human/mouse organoid culture to mimic intestinal epithelium within its cell type diversity represent a unique tool to address specific STm/IEC interaction. But it requires *in vivo* confirmation including the complex environment present in the intestine. Regarding intestinal organoid culture challenges remain now to establish reproducibility, lower the cost and increase high throughput.

## Author Contributions

SM and AW outlined the manuscript. SM, SL-L, KE, JY, GG, and AW wrote and critically revised the manuscript. SM and JY prepared the figures. All authors read and approved the final version of the article.

## Funding

The funding from the Région Occitanie/FEDER within “Nanorgan” project, collection COLIC, CHU Toulouse, France and the Deutsche Forschungsgemeinschaft (DFG, German Research Foundation) – SFB900/3 – 158989968, TP08, and by the German Center for Infection Research (DZIF), partner site Hannover-Braunschweig.

## Conflict of Interest

The authors declare that the research was conducted in the absence of any commercial or financial relationships that could be construed as a potential conflict of interest.

## Publisher’s Note

All claims expressed in this article are solely those of the authors and do not necessarily represent those of their affiliated organizations, or those of the publisher, the editors and the reviewers. Any product that may be evaluated in this article, or claim that may be made by its manufacturer, is not guaranteed or endorsed by the publisher.

## Abbreviations

AMP: Anti-microbial peptides
3D: 3-Dimension
EGFR: Epidermal growth factor receptor
GP2: Glycoprotein 2
IL: Interleukin
Lgr5^+^: Leu-rich repeat containing G protein-coupled receptor 5
M cells: Microfold
MMP: Matrix metalloproteinases
PagN: PhoP activated gene N
PP: Peyer’s patches
pIgR: Immunoglobulin receptor
Rck: Resistance to complement killing
SCV: *Salmonella* containing vacuole
SIgA: Secretory IgA
Sif: *Salmonella* induced filament
Sip: *Salmonella* invasion protein
Sop: *Almonella* outer protein
STm: *Salmonella* Typhimurium
T3SS: Type III secretion system
