# Supplementary material for: Cross-Talk Between the Intestinal Epithelium and Salmonella Typhimurium
Source: Front Microbiol. 2022 Jun 6;13:906238. doi: 10.3389/fmicb.2022.906238 (PMC9207452; doi:10.3389/fmicb.2022.906238)
Supplement: Supplementary file 1 [file Table_1.DOCX]

Table 1: Examples for members of the different intestinal protease classes expressed in the naïve and/or *Salmonella*-infected intestine.

| Serine proteases | Elastase, Matriptase  Plasmin, Thrombin  Kallikrein (5, 8 and 22), Trypsin, Chymotrypsin  Chymase, Tryptase |
| --- | --- |
| Cysteine proteases | Caspases  lysosomal Cathepsins K |
| Aspartate proteases | Cathepsin E |
| Metalloproteinase | Matrix metalloproteinases (MMPs) (2,3, 7, 8, 9 and 10) |
| Threonine proteases | in proteasome |
